# Bioactives of the essential oil from the leaves of *Eugenia pyriformis* Cambess (Myrtaceae) on the effects of tobacco

**DOI:** 10.3389/fphar.2024.1415659

**Published:** 2024-06-07

**Authors:** Jaqueline Pavelegini de Medeiros, Selma Alves Rodrigues, Karina Sakumoto, Suelen Pereira Ruiz, Maria Graciela Iecher Faria, José Eduardo Gonçalves, Ranulfo Piau Junior, Jasmina Glamočlija, Marina Soković, Daniela Dib Gonçalves, Filipa Mandim, Lillian Barros, Zilda Cristiani Gazim

**Affiliations:** ^1^ Graduate Program in Biotechnology Applied to Agriculture, Universidade Paranaense, Umuarama, Brazil; ^2^ Graduate Program in Animal Science With Emphasis on Bioactive Products, Universidade Paranaense, Umuarama, Brazil; ^3^ Graduate Program in Medicinal and Phytotherapeutic Plants in Primary Care, Universidade Paranaense, Umuarama, Brazil; ^4^ Graduate Program in Clean Technologies, UniCesumar, Maringá, Brazil; ^5^ Cesumar Institute of Science, Technology and Innovation (ICETI), Maringá, Brazil; ^6^ Institute for Biological Research “Sinisa Stankovic”, National Institute of Republic of Serbia, University of Belgrade, Belgrade, Serbia; ^7^ Laboratório Associado para a Sustentabilidade e Tecnologia em Regiões de Montanha (SusTEC), Instituto Politécnico de Bragança, Campus de Santa Apolónia, Bragança, Portugal; ^8^ Centro de Investigação de Montanha (CIMO), Instituto Politécnico de Bragança, Campus de Santa Apolónia, Bragança, Portugal

**Keywords:** lung carcinoma, antiproliferative, uvaia, nitric oxide, antimicrobial activity, β-caryophyllene oxide, spathulenol

## Abstract

**Introduction:**

Lung cancer is the most commonly diagnosed and the main cause of cancer death, usually related to cigarette smoking. Furthermore, the microbiota of people exposed to cigarette smoke can be modified, making it difficult to eliminate opportunistic microorganisms. The leaves of *Eugenia pyriformis* are a by-product of fruit production and, to date, there have been no studies addressing the antiproliferative, anti-inflammatory, and antimicrobial activities.

**Objective:**

Investigate the antimicrobial, Nitric Oxide (NO)-production inhibition, and antiproliferative activities of the essential oil from *E. pyriformis* leaves and its possible effect on the treatment and prevention of damage caused by tobacco.

**Methods:**

The essential oil (EO) was obtained by hydrodistillation (3 h). Its chemical composition was investigated by GC-MS. It was proposed to investigate antiproliferative activity against human tumor cell lines, namely, breast adenocarcinoma (MCF-7), lung (NCI-H460), cervical (HeLa), and hepatocellular (HepG2) carcinomas. A non-tumor primary culture from pig liver (PLP2) was also tested. The EO capacity to inhibit nitric oxide (NO) production was evaluated by a lipopolysaccharide stimulated murine macrophage cell line. Antibacterial and antifungal activities against opportunistic pathogens were investigated against seven strains of bacteria and eight fungi.

**Results:**

The results indicated the presence of 23 compounds in the essential oil, the majority were spathulenol (45.63%) and β-caryophyllene oxide (12.72%). Leaf EO provided 50% inhibition of nitric oxide production at a concentration of 92.04 µg mL^−1^. The EO also demonstrated antiproliferative activity against all human tumor cell lines studied, with GI50 values comprised between 270.86 and 337.25 µg mL^−1^. The essential oil showed antimicrobial potential against the bacteria *Listeria* monocytogenes (Murray et al.) Pirie (NCTC 7973) and *Salmonella Typhimurium* ATCC 13311 (MIC 1870 µg mL^−1^) and fungi *Aspergillus versicolor* ATCC 11730, *Aspergillus ochraceus* ATCC 12066, *Penicillium ochrochloron* ATCC 90288, *Penicillium verrucosum* var. cyclopium (Westling) Samson, Stolk & Hadlok (food isolate) (MIC 1870 µg mL^−1^) and *Trichoderma viride* Pers. IAM 5061 (1,400 µg mL^–1^).

**Conclusion:**

The demonstrated anti-inflammatory, antiproliferative, and antimicrobial activities in the leaves of *E. pyriformis* can add value to the production chain of this plant, being a possible option for preventing and combating cancer, including lung cancer.

## 1 Introduction

The World Health Organization (WHO) named tobacco as one of the biggest public health problems of the 21st century (Das and Gupta, 2021). The high rates of morbidity and mortality associated with exposure to tobacco smoke are due to the cytotoxic, mutagenic, genotoxic, and carcinogenic effects of cigarette smoke constituents and combustion products (Sołtysiak et al., 2024). Tobacco releases a complex mixture of more than 7,000 chemical constituents characterized by a gaseous mixture of irritating and toxic gases (carbon monoxide, volatile organic compounds, SO, NO) and a solid residue, consisting of fine dust and fine and ultrafine particles (Di Giacomo et al., 2018), including carcinogens, oxidants, and toxins that can initiate inflammatory pathways that lead to smoking-related diseases (Addissouky et al., 2024).

Chemical substances from tobacco cause a series of physiological changes. Smoking is associated with gastrointestinal disorders and has relevant effects on delaying gastric emptying. The action of nicotine and other tobacco constituents is capable of altering the binding sites of plasma proteins. Cigarette smoke contains several chemical constituents, including polycyclic aromatic hydrocarbons, which are mainly responsible for altering liver enzymes. The pathophysiological changes present in smoking can affect the absorption, distribution, metabolism, and excretion processes (de Sousa et al., 2018).

Smoke derivatives released into the environment are considered by the International Agency for Research on Cancer to pose an important carcinogenic risk to humans (Di Jacomo et al., 2008). Another factor is that ex-smokers continue to have a high risk of cancer for years after quitting smoking: more than half of lung cancers occur in those who have stopped smoking (Izano et al., 2015). The proposal to investigate the protective effect of natural compounds, with emphasis on essential oils, on cells subjected to cigarette smoke, shows the importance of these compounds in the effects resulting from tobacco.

Another effect of tobacco is microbiota dysbiosis, as the substances present cause disruption of microbial homeostasis with compositional or functional deviation. It is known that the microbiota participates in several physiological processes, including digestion, metabolism, immune system, and defense against pathogen invasion. Luminal microorganisms can affect cancer progression by altering the permeability of the mucosal barrier, activating inflammatory pathways, producing bacterial toxins that impair host genome stability, releasing cancer-promoting metabolites, and modulating the local immune microenvironment (Zheng et al., 2023). In this way, tobacco contributes to changes in the oral, lung, and intestinal microbiome, leading to several diseases, such as periodontitis, asthma, chronic obstructive pulmonary disease (COPD), Crohn’s disease, ulcerative colitis, and cancer (Huang and Shi, 2019).

The main types of cancer resulting from smoking are lung and oral cancer (Das and Gupta, 2021). Lung cancer remains the leading cause of cancer-related deaths worldwide, and one of the three most common, only surpassed by prostate and breast cancer in women. For the year 2022, 1,918,030 new cases of cancer were estimated in the United States. Of these, lung cancer contributes 236,740 (12.3%) of new cases with shares of 117,910 and 118,830 for men and women, respectively. In Brazil, according to 2020 estimates, lung cancer is the third most common in men (17,760 new cases) and the fourth in women (12,440 new cases) (Paschoal, 2023).

The main treatment options for lung cancer include surgery, radiotherapy, chemotherapy, driver gene-targeted therapy, and the recently emerged immunotherapy (Zheng et al., 2023). Another form of treatment and prevention of the effects caused by cigarettes concerns medicinal plants and compounds isolated from plants. Garlic extracts (*Allium sativum* L.), artichoke preparations (*Cynara scolymus* L.) or statin derivatives (e.g., Lovastatin), which was originally derived from the fungus *Aspergillus terreus*, were tested *in vivo* and *in vitro* and measured their potential to protect and reverse degenerative changes in artery walls caused by cigarette smoking (Schmieder et al., 2007). In addition to these, essential oils from the Myrtaceae family, such as *Psidium myrtoides* (Dias et al., 2019), *Myrciaria tenella* (Ferreira et al., 2024), and *Eugenia pyriformis* (Durazzini et al., 2019) have been demonstrated potential activity antiproliferative.

*Eugenia pyriformis* Cambess (Myrtaceae) is a tree native to Brazil, commonly found in the pine forests of southern Brazil, as well as in Argentina and Paraguay (Sganzerla et al., 2021). Popularly known as *uvaia*, *uvaieira*, *uvaia-do-campo*, *uvalha* or *uvalha-do-campo*, this plant can be used in reforestation programs and urban areas, also cultivated in domestic orchards, for consumption of the fruits in form of juices and jellies, presenting the potential for industrial use (Stefanello et al., 2009).

Previous studies with the essential oil of *E*. *pyriformis* showed acaricidal action from leaves (Medeiros et al., 2019), antileishmanial, antiproliferative from leaves and twigs (aerial parts) (Durazzini et al., 2019) and antimicrobial and antioxidant from fruits (Stieven et al., 2009). However, studies on possible pharmacological properties of the essential oil from *E*. *pyriformis* leaves are still scarce. Due to this and the possibility of verifying new biological properties, the aim of the present work was to investigate the antimicrobial, anti-inflammatory, and antiproliferative activities of the essential oil from *E*. *pyriformis* leaves and its possible effect on the treatment and prevention of damage caused by tobacco.

## 2 Material and methods

### 2.1 Plant material

Leaves of *E. pyriformis* were collected in the vegetative phenological phase in the municipality of Mangueirinha, southwestern Paraná state, Brazil at latitude 25°56′28″ S; 52°10′32″ W), altitude of 921 m, between May and July 2016. A plant sample was authenticated and deposited in the Herbarium of the *Universidade Estadual de Maringá* (HUEM) under the number 30713. The plant was registered in the National System for the Management of Genetic Heritage and Associated Traditional Knowledge (SisGen, an acronym in Portuguese) under the registration number A3DD0FF.

### 2.2 Essential oil extraction

To extract the essential oil, the leaves were dried at room temperature. Leaves of *E. pyriformis* were fragmented with water (1:10) in an industrial blender for 5 min and immediately submitted to hydrodistillation in a modified Clevenger apparatus for 3 h. After the distillation, the essential oil was stored in an amber flask at −4°C (Apel et al., 2004; Stefanello et al., 2009) with modifications (Medeiros et al., 2019). EO yield was determined by the dry leaf mass to EO mass (%) ratio.

### 2.3 Gas chromatography coupled to mass spectrometry

The identification of the chemical constituents of the essential oil from *E*. *pyriformis* leaves occurred by gas chromatography (GC, Agilent^®^ Technologies 7890B, Santa Clara, United States) coupled to mass spectrometry (MS, Agilent^®^ Technologies 5977A, Santa Clara, United States). The compounds were separated using an HP-5MS-UI capillary column (30 m × 0.25 mm × 0.25 μm). The column temperature was from 50°C (2 min) to 60°C (1 min) with a ramp of 5°C/min, to 250°C with a ramp of 5°C/min, remaining at 250°C for 15 min and finishing to 300°C (1 min) with a ramp of 50°C/min. The carrier gas was helium, used at a constant pressure of 80 kPa and a linear speed of 1 mL min^−1^ up to 210°C and a pressure flow rate of 52.74 kPa. Injection in split mode (1:20) with injector temperature of 250°C. The mass conditions (MS) were source temperature 230°C; quadrupole temperature 150°C; interface temperature 250°C; 70 eV detector; mass scan range, 40–550 amu (Stefanello et al., 2009). In addition to the results obtained by GC-MS, the identification of the compounds was also based on the comparison of the retention indices (RI) obtained using a homologous series of n-alkanes (C7–C40). The mass spectra were compared with the Wiley 275 spectrum library and with the literature (Adams, 2017).

### 2.4 Antimicrobial activity

#### 2.4.1 Antibacterial activity

The antibacterial activity of *E. pyriformis* essential oil was tested against three Gram-positive strains such as *Bacillus cereus* Frankland & Frankland (clinical isolate), *Listeria monocytogenes* (Murray et al.) Pirie (NCTC 7973), and *Staphylococcus aureus* subsp. *aureus* Rosenbach (ATCC 6538), and four Gram-negative strains as *Enterobacter cloacae* (Jordan) Hormaeche & Edwards (clinical isolate), *Escherichia coli* (Migula) Castellani & Chalmers (ATCC 35218), *Pseudomonas aeruginosa* (Schroeter) Migula (ATCC 27853), and *Salmonella enterica* subsp. *enterica* (ex Kauffmann & Edwards) Le Minor & Popoff serovar Typhimurium (ATCC 13311). Antibacterial assays were performed by the broth microdilution method (CLSI, 2009) in 96-well microplates. Bacterial suspensions were standardized with sterile saline solution to a concentration of 1.0 × 10^5^ colony-forming units (CFU) mL^−1^ (Hänel and Raether, 1988; Espinel-Ingroff, 2001). The essential oil was dissolved in a 5% dimethyl sulfoxide solution (Merck KGaA, Germany) containing 0.1% of polysorbate-80 (1 mg mL^−1^) and added to Luria-Bertani (100 µL) medium with bacterial inoculum (1.0 × 10^4^ CFU well^−1^) to reach the desired concentrations. The microplates were incubated in a rotary agitator (160 rpm) for 24 h at 37°C. The minimum inhibitory concentration (MIC) was defined as the lowest concentration without visible bacterial growth under an optical microscope. The minimum bactericidal concentration (MBC) was determined by serial subcultivation of 2 μL inoculum in microtiter plates containing 100 μL LB per well, followed by incubation for 24 h at 37°C. The lowest essential oil concentration without visible bacterial growth under an optical microscope was defined as the MBC, indicating 99.5% killing of the original inoculum. The optical density of each well was measured at 655 nm using a microplate reader equipped with Microplate Manager software version 4.0 (Bio-Rad^®^ Laboratories, Hercules, CA, United States). An aqueous solution of DMSO (50 mL L^−1^) was used as a negative control. The commercial antibiotics streptomycin (Sigma P7794, San Luis, MO, United States) and ampicillin (Panfarma, Belgrade, Serbia) (1 mg mL^−1^) were used as positive control.

#### 2.4.2 Antifungal activity

Antifungal property of the essential oil was tested against seven fungal strains such as *Aspergillus fumigatus* Fresenius (ATCC 1022), *Aspergillus ochraceus* Batista & Maia (ATCC 12066), *Aspergillus niger* van Tieghem (ATCC 6275), *Aspergillus versicolor* (Vuillemin) Tiraboschi (ATCC 11730), *Penicillium funiculosum* Thom (ATCC 8725), *Penicillium ochrochloron* Biourge (ATCC 90288), *Penicillium verrucosum* var. cyclopium (Westling) Samson, Stolk & Hadlok (food isolate) and *Trichoderma viride* Pers. (IAM 5061). Antifungal activity was assessed by the modified microdilution method (Hänel and Raether, 1988; Espinel-Ingroff, 2001). Fungal spores were washed from agar plate surfaces with an aqueous sterile solution containing 8.5 mg mL^−1^ NaCl and 1.0 mg mL^−1^ polysorbate-80. The spore suspension was adjusted to 1.0 × 10^5^ spores in 100 μL solution per well. The MIC of essential oil was determined by serial dilution in 96-well microtiter plates. The essential oil of *E. pyriformis* was diluted (100–100,000 μg mL^−1^) in an aqueous solution containing 50 mL L^−1^ DMSO and 1 mg mL^−1^ polysorbate-80 and added to malt extract broth with inoculum. The microplates were incubated in a rotary agitator (160 rpm) for 72 h at 28°C. MIC was defined as the lowest concentration of essential oil that completely inhibited fungal growth, as assessed under an optical microscope. The minimum fungicidal concentration (MFC) was determined by serial subcultivation using 2 μL broth per well in microplates containing 100 μL malt extract broth followed by incubation at 28°C for 72 h. MFC was defined as the lowest concentration without visible growth, indicating 99.5% killing of the original inoculum. An aqueous solution of DMSO (50 mL L^−1^) was used as a negative control. The commercial fungicides bifonazole (Srbolek, Belgrade, Serbia) and ketoconazole (Zorkapharma, Šabac, Serbia) were used as positive control.

### 2.5 NO-production inhibition assay

The capacity of *E. pyriformis* leaves essential oil to inhibit nitric oxide (NO) production was evaluated according to the procedure previously described by Corrêa et al. (2015). The essential oil was dissolved in DMSO in order to obtain a final concentration of 8 mg mL^−1^. Successive dilutions were carried out with H_2_O, obtaining the concentrations to be tested (0.125–8 mg mL^−1^). The cell line of murine macrophages (RAW 264.7) was commercially obtained from the European Collection of Authenticated Cell Cultures (ECACC). This cell line was cultivated in Dulbecco’s modified eagle’s medium (DMEM) (HyClone, Logan, Utah, United States), supplemented with 10% of bovine fetal serum (FBS) inactivated by heat, glutamine (2 mM), penicillin (100 U mL^−1^) and streptomycin (100 mg mL^−1^) (HyClone, Logan, Utah, United States) and maintained at 37°C, with 5% CO₂ and in humidified air (Heal Force CO_2_ Incubator, Shanghai Lishen Scientific Equipment Co., Ltd.). The cells were detached with a cell scraper and a solution with a density of 5 × 10⁵ cells mL^−1^ was prepared and transferred to 96-well plates. After incubation for 24 h, the cells were treated with the different concentrations of essential oil (15 μL, final concentrations tested between 6.25 and 400 μg mL^−1^) for an hour and were stimulated with lipopolysaccharide (LPS) (1 μg mL^−1^) (Sigma, St. Louis, MO, United States) for 24 h. The assay to evaluate the capacity of the studied samples to inhibit NO production was developed after confirming that the concentrations tested were non-toxic to the cells. This assay was carried out with the colorimetric assay of sulforhodamine B. Dexamethasone (50 µM) was used as a positive control, and the cells in the presence and absence of LPS were used as negative controls.

The determination of nitric oxide was performed according to Corrêa et al. (2015), utilizing a Griess reagent kit (Promega Corporation, Madison, WI, United States) that contains sulfanilamide, N-(1-naphthyl) ethylene diamine hydrochloride (NED) and nitrite solutions. A nitrite reference curve (sodium nitrite from 100 µM to 1.6 µM; y = 0.0068x + 0.0951; *R* ^2^ = 0.9864) was prepared in a 96-well plate. The supernatant of the cell culture (100 µL) was transferred to the plate and mixed with Griess reagent from 5 to 10 min at room temperature. The produced nitrite was determined by absorbance measurement at 540 nm (microplate reader ELX800 Biotek) and by comparison to the standard calibration curve. The results were expressed in EC₅₀ which corresponds to the effective concentration providing 50% of inhibition of nitric oxide production and values expressed in μg mL^−1^.

### 2.6 Antiproliferative activity

The antiproliferative activity of *E. pyriformis* leaves essential oil was investigated against four human tumor cell lines: breast adenocarcinoma (MCF-7), lung (NCI-H460), cervical (HeLa), and hepatocellular (HepG2) carcinomas. A non-tumor primary culture from pig liver (PLP2) was also tested. All the cell lines studied were routinely kept as adherent cell cultures in Gibco Roswell Park Memorial Institute (RPMI-1640) medium supplemented as described above (Section 2.5). Each cell line was plated at a density of 1.0 × 10⁴ cells per well in 96-well plates. The colorimetric assay of sulforhodamine B (Extra synthèse, Genay, France) was conducted according to the procedure described by Barros et al. (2013). Ellipticine was used as positive control and the cells in the absence of samples as negative control. The obtained results were expressed as the concentration responsible for 50% cell proliferation inhibition (GI_50_ values, μg mL^−1^).

### 2.7 Statistical analysis

Results of the antimicrobial assays are presented as arithmetic average ± standard deviation (*n* = 6). Data were analyzed by one-way analysis of variance (ANOVA) followed by the Scott–Knott test (*p* ≤ 0.05) for comparison of means. Analyses were performed using Statistical Package for the Social Sciences (SPSS) v 18.0 (Statistics, Armonk, NY, United States). The results of antiproliferative activity and NO-production inhibition assay are expressed in arithmetic average values ± standard deviation (*n* = 3) and analyzed by ANOVA, followed by Tukey’s HSD (honestly significant difference) test at α = 0.05 by SPSS v. 22.0.

## 3 Results

### 3.1 Chemical composition of the essential oil

**FIGURE 1:**
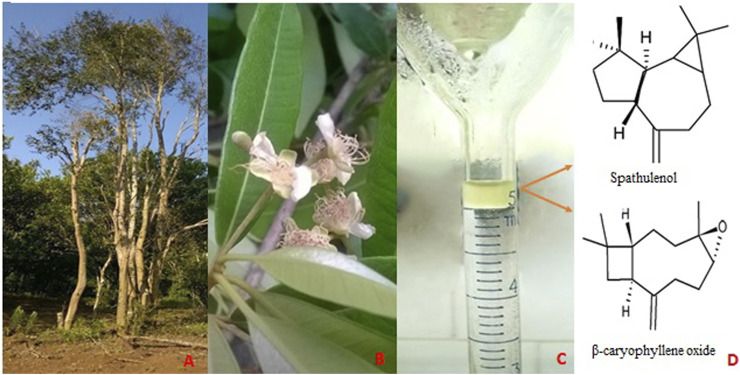
**(A)** Adult specimen of *Eugenia pyriformis*; **(B)** *Eugenia pyriformis* leaves and flowers; **(C)** *Eugenia pyriformis* leaves essential oil; **(D)** Chemical structure of the main major compounds identified of *Eugenia pyriformis* leaves essential oil. Source: The Authors.

The yield of *E. pyriformis* leaves essential oil obtained by hydrodistillation was 0.029%. The results of the chemical analysis of the essential oil extracted from *Eugenia pyriformis* leaves are shown in Table 1, showing that oxygenated sesquiterpenes were the majority class (84.10%), and Spathulenol (45.63%) and β-caryophyllene oxide (12.72%) as major compounds (Figure 1).

**TABLE 1:** Chemical composition of *Eugenia pyriformis* Cambess leaves essential oil by gas chromatography coupled to mass spectrometry (GC/MS).

Peak	Compounds	Relative area (%)	RI calculated	RI literature	IM
Hydrocarbon Sesquiterpenes					
1	α-copaene	1.16	1,376	1,374	b,c,d
2	β-bourbonene	2.35	1,388	1,387	,b,c,d
3	β-caryophyllene	1.56	1,419	1,417	b,c,d
4	Aromadendrene	1.46	1,441	1,439	b,c,d
5	α-humulene	1.23	1,454	1,452	b,c,d
6	Alloaromadendrene	2.48	1,460	1,458	b,c,d
7	Germacrene D	t	1,485	1,484	b,c,d
8	Bicyclogermacrene	2.08	1,500	1,500	b,c,d
9	*γ*-cadinene	1.11	1,513	1,513	b,c,d
10	*α*-cadinene	1.31	1,538	1,537	b,c,d
Oxygenated sesquiterpenes					
11	Spathulenol	45.63	1,578	1,577	b,c,d
12	Caryophyllene oxide	12.72	1,583	1,582	b,c,d
13	Viridiflorol	5.24	1,592	1,592	b,c,d
14	Globulol	1.94	1,590	1,590	b,c,d
15	Guaiol	2.46	1,596	1,600	b,c,d
16	Ledol	2.93	1,602	1,602	b,c,d
17	Isoaromadendrene epoxide	1.06	1,617	1,612	b,c,d
18	Isospathulenol	1.62	1,625	1,628	b,c,d
19	*epi*-*α*-cadinol	4.48	1,638	1,638	b,c,d
20	Alloaromadendrene epoxide	1.61	1,641	1,639	b,c,d
21	*epi*-*α*-muurolol	4.37	1,640	1,640	b,c,d
22	Caryophylla-3,8(13)-dien-5β-ol	t	1,640	1,649	b,c,d
23	*α*-santalol	t	1,674	1,674	b,c,d
24	*β*-santalol	1.10	1715	1,715	b,c,d
	Total identified	98.84			
Hydrocarbon Sesquiterpenes		14.74			
Oxygenated sesquiterpenes		84.10			
Not identified		1.06			

Compounds listed in elution order through the capillary column HP5. ^b^RI Calculated: The retention index (RI) was calculated using a homologous n-alkane series C7 to C40 in the capillary column (HP5). ^c^The identification of compounds was based on the comparison of mass spectra to Wiley 275 spectrum library. ^d^literature retention index (RI): Adams, 2017 and Silva, Pott, et al. (2010) for Isospathulenol; Lawal and Oyedelji (2009) for Caryophylla-3,8(13)-dien-5β-ol; Relative area (%): percentage of the area occupied by compounds within the chromatogram; n.i.: non-identified; t: traces; IM: identification methods. For the analysis, only compounds with a relative area (%) greater than 1.0% were considered.

### 3.2 Antimicrobial activity

The results of the antimicrobial activity of *E*. *pyriformis* essential oil are shown in Table 2. The lowest MIC values were for *L*. *monocytogenes* and *Salmonella Typhimurium* (1,870 μg mL^−1^). Regarding MBC values for the essential oil, they were 3,750 μg mL^−1^ for all species of bacteria (Table 2).

**TABLE 2:** Minimum Inhibitory Concentration (MIC), Minimum Bactericidal Concentration (MBC) and Minimum Fungicide Concentration (MFC) of Essential Oil from *Eugenia pyriformis* leaves (μg mL^−1^) in bacterial strains.

	*Eugenia pyriformis* (μg mL^−1^)		Streptomycin (μg mL^−1^)		Ampicillin (μg mL^−1^)	
	MIC	MBC	MIC	MBC	MIC	MBC
*Bacillus cereus*	2,810 ± 400^bB^	3,750 ± 300^aB^	100 ± 3^bA^	200 ± 60^bA^	250 ± 4^aA^	400 ± 30^aA^
*Listeria* monocytogenes	1,870 ± 200^aB^	3,750 ± 300^aB^	200 ± 30^cdA^	300 ± 0^cA^	400 ± 0^bA^	500 ± 20^aA^
*Micrococcus flavus*	2,810 ± 300^bB^	3,750 ± 100^aB^	200 ± 30^cdA^	300 ± 10^cA^	250 ± 60^aA^	400 ± 0.0^aA^
*Staphylococcus aureus*	2,810 ± 400^bB^	3,750 ± 300^aB^	40 ± 2^aA^	100 ± 2^aA^	250 ± 60^aA^	400 ± 10^aA^
*Escherichia coli*	2,810 ± 400^bB^	3,750 ± 400^aB^	200 ± 20^cdA^	300 ± 10^cA^	400 ± 20^bA^	500 ± 60^aA^
*Enterobacter cloacae*	2,810 ± 0^bC^	3,750 ± 600^aB^	200 ± 2^cdA^	300 ± 40^cA^	250 ± 30^aB^	500 ± 40^aA^
*Pseudomonas aeruginosa*	2,810 ± 400^bB^	3,750 ± 0^aC^	194 ± 12.2^cA^	300 ± 0^cA^	750 ± 30^cA^	1,200 ± 200^cB^
*Salmonella* Typhimurium	1,870 ± 300^aB^	3,750 ± 200^aB^	250 ± 20^dA^	500 ± 20^dA^	400 ± 20^bA^	750 ± 20^bA^

	*Eugenia pyriformis* (μg mL^−1^)		Bifonazole (μg mL^−1^)		Ketoconazole (μg mL^−1^)	
	MIC	MFC	MIC	MFC	MIC	MFC
*Aspergillus fumigatus*	5,620 ± 300^cB^	3,750 ± 300^aB^	150 ± 20^abA^	200 ± 60^bA^	200 ± 20^aA^	400 ± 30^aA^
*Aspergillus versicolor*	1,870 ± 100^aB^	3,750 ± 300^aB^	100 ± 0^aA^	300 ± 0^cA^	200 ± 60^aA^	500 ± 20^aA^
*Aspergillus ochraceus*	1,870 ± 100^aC^	3,750 ± 100^aB^	150 ± 30^abA^	300 ± 10^cA^	1,500 ± 200^cB^	400 ± 0^aA^
*Aspergillus niger*	7,500 ± 600^dB^	3,750 ± 300^aB^	150 ± 40^abA^	100 ± 2^aA^	200 ± 20^aA^	400 ± 10^aA^
*Penicillium funiculosum*	3,750 ± 0^bB^	3,750 ± 400^aB^	200 ± 20^bA^	300 ± 10^cA^	200 ± 20^aA^	500 ± 60^aA^
*Penicillium ochrochloron*	1,870 ± 200^aB^	3,750 ± 600^aB^	200 ± 10^bA^	300 ± 40^cA^	2,500 ± 300^dC^	500 ± 40^aA^
*Penicillium verrucosum*	1,870 ± 200^aB^	3,750 ± 0^aC^	100 ± 20^aA^	300 ± 0^cA^	200 ± 30^aA^	1,200 ± 200^cB^
*Trichonoderma viride*	1,400 ± 200^aB^	3,750 ± 200^aB^	150 ± 30^abA^	500 ± 20^dA^	100 ± 200^bB^	750 ± 20^bA^

Values expressed as arithmetic mean ± standard deviation. The means followed by different uppercase letters on the same line for MIC and different lowercase letters in column for MBC or MFC show a significant difference by Tukey test (*p* ≤ 0.05).

For the fungi studied, the best results were observed against *T*. *viride* (1,400 μg mL^−1^) and *A*. *versicolor*, *A*. *ochraceus*, *P*. *ochrochloron*, and *P*. *verrucosum*, all with MIC of 1,870 μg mL^−1^. Regarding MFC, the value was 3,750 μg mL^−1^ for all fungi (Table 2).

### 3.3 NO-production inhibition assay

The results of the NO-production inhibition capacity of *E. pyriformis* essential oil are shown in Table 3. The essential oil inhibited the production of nitric oxide in RAW 264.7 macrophages stimulated by lipopolysaccharide (LPS) with an EC₅₀ (effective concentration providing 50% inhibition of nitric oxide production) of 92.04 μg mL^−1^, being only 5.86 times less active than the positive control dexamethasone (EC₅₀ = 15.70 μg mL^−1^).

**TABLE 3:** Antiproliferative and NO-production inhibition activity of *Eugenia pyriformis* leaves essential oil.

Antiproliferative activity (GI_50_ values, μg mL^−1^)				
	Essential oil	SI	Ellipticine	SI
Breast adenocarcinoma (MCF-7)	291.51 ± 19.56^aB^	1.29	0.91 ± 0.04^aA^	3.54
Lung carcinoma (NCI-H460)	328.00 ± 15.55^abB^	1.15	1.03 ± 0.09^aA^	3.13
Cervical carcinoma (HeLa)	337.25 ± 18.24^bB^	1.12	1.91 ± 0.06^aA^	1.70
Hepatocellular carcinoma (HepG2)	270.86 ± 27.02^aB^	1.40	1.14 ± 0.21^aA^	2.82
Porcine liver primary cells (PLP2)	378.50 ± 9.65^aB^	-	3.22 ± 0.67^aA^	-

Murine macrophage cells (RAW 246.7)				
	Essential oil		Dexamethasone	
Toxic effects (GI_50_ values, μg mL^−1^)	>400			
NO-production inhibition (EC_50_, μg mL^−1^)	92.04 ± 8.24^b^		15.70 ± 1.10^a^	

Values are expressed as arithmetic mean ± standard deviation. GI₅₀, essential oil concentration responsible for 50% cell proliferation inhibition; SI, selectivity index (GI_50_ μg mL^−1^ non-tumor cell line/GI_50_ μg mL^−1^ tumor cell line). EC₅₀, effective concentration providing 50% inhibition of nitric oxide (NO) production. The means followed by the same uppercase letters in the lines and the same lowercase letters in the column do not differ significantly by Tukey’s HSD test (*p* ≤ 0.05).

### 3.4 Antiproliferative activity

The results of the antiproliferative activity in tumor and non-tumor cells and the selectivity index of the essential oil from E. pyriformis leaves are shown in Table 3. There was inhibition in the proliferation of the four human tumor cell lines tested, with GI_50_ values ranging between 270.86 and 337.25 μg mL^−1^ (Table 3). Concerning the non-tumor primary culture, obtained from pig liver, the essential oil shows a low capacity to interfere with these cells’ proliferation (GI_50_ = 378.50 μg mL^−1^) and, when compared with the commercial ellipticine (GI_50_ = 3.22 μg mL^−1^), the essential oil was 117.54 times less toxic (Table 3).

With the GI_50_ values obtained, it was also possible to establish selectivity indices (SI), being greater than 1, which shows the relationship between the GI_50_ of tumor cells and non-tumor cells and can be used to estimate the safety of a compound.

## 4 Discussion

In the essential oil of *E*. *pyriformis* leaves the major compounds were spathulenol (45.6%), caryophyllene oxide (12.7%), viridiflorol (5.2%), epi-α-cadinol (4.5%) and epi-α-muurolol (4.4%) (Table 1). Spathulenol, a sesquiterpene reported to be major volatile component of the essential oils of several aromatic Myrtaceae species, including *Eugenia calycina* Cambess., *E. Uniflora* L., *Psidium guajava* L. and *P. cattleianum* Afzel. ex Sabine (do Nascimento et al., 2018).

In the present study, the antimicrobial activity of uvaia essential oil was evaluated (Table 2), It is worth highlighting that both active smoking and passive exposure to smoke can lead to increased colonization of the body by pathogenic bacteria (Huang and Shi, 2019). Furthermore, mechanisms related to smoking influence the microbiota through changes in homeostasis, biofilm formation, and oxygen tension, and these mechanisms may be involved in the occurrence of diseases, not only oral and pulmonary but also intestinal (Huang and Shi, 2019).

The essential oil showed antimicrobial activity on all species of bacteria and fungi evaluated in the present study. Antimicrobial activities may be associated with the major compounds present in the essential oil in this study. Regarding antibacterial activity, the greater antibacterial potential was against *Listeria monocytogenes* and *Salmonella Typhimurium*. Souza et al. (2021) reported that essential oil from the leaves of *E. pyriformis* β-Caryophyllene (17.82%), Bicyclogermacrene (12.84%), Globulol (5.96%) and δ-Cadinene (4.33%)) showed MIC values of 250 and 125 μg mL^−1^ against *S. aureus* and methicillin-resistant *S. aureus* (MRSA), respectively. β-caryophyllene, one of the majority in the present study, was reported activity on Gram-negative and Gram-positive bacteria (Dahham et al., 2015), with MIC values for *S. aureus* (32 μg mL^−1^) (Santos et al., 2021), *S. typhimurium* (500 μg mL^−1^) and *E. coli* (10,000 μg mL^−1^) (Neta et al., 2017). Spathulenol, the main compound also present in this study, presented MIC values for *Enterococcus faecalis* (200 μg mL^−1^), but at the maximum concentration evaluated (200 μg mL^−1^), it did not show activity against *S. aureus*, *P. aeruginosa*, and *E. coli* (Fernandez et al., 2015).

With these results, it can be suggested that the antibacterial activity of uvaia essential oil may be related to the synergy of the compounds. It is known that tobacco smoke affects the virulence of *S*. *aureus* (McEachern et al., 2015), in addition to increasing binding to epithelial cells in the oral cavity (Hwang et al., 2016). Therefore, bacterial infection can provide conditions for chronic inflammation, being one of the main factors related to oral cancer (Zhang et al., 2019). Additionally, tobacco may have an impact on antibiotic prescriptions, as people who smoke are prescribed more antibiotics than non-smokers (Steinberg et al., 2016). Therefore, the use of natural substances such as essential oils with antibacterial action, as in the present study, could help to alleviate these effects.

The essential oil from uvaia leaves showed potential antifungal activity with MIC ranging between 1,400 and 7,500 μg mL^−1^ against the fungi tested (Table 2). Tobacco use can also facilitate lung infections caused by filamentous fungi. Alighiri (2022) studied the antifungal activity of caryophyllene, which at a concentration of 1.2% showed activity against *A. fumigatus*, using the agar diffusion method. Neta et al. (2017) verified the capacity of β-caryophyllene against *A. niger* and *A. fumigatus* with an MIC of 500 μg mL^−1^.

Dermawan, Ghosh and Mukhopadhyay (2020) studied a subset of aspergillosis characterized by radiologically solid pulmonary nodules, with eight patients classified with these characteristics. All were smokers or ex-smokers with a history of chronic obstructive pulmonary disease (COPD) and evidence of emphysema, none of the patients were immunocompromised and only two of them were able to identify the species involved: *A. niger* and *A. fumigatus.* The treatment of pulmonary aspergillosis is complicated by the limited number of antifungal options, drug interactions, adverse events, and the emergence of antifungal resistance (Lamoth and Calandra, 2022). Therefore, the use of uvaia leaf essential oil could be a new treatment option in the near future.

Regarding the NO-production inhibition, the essential oil of *E*. *pyriformis* exhibited the capacity to inhibit the production of the pro-inflammatory mediator, nitric oxide in a murine macrophage (RAW 264.7), stimulated by lipopolysaccharide (Table 3). This result suggests the promising activity of the studied essential oil, endowed with a rich chemical composition. Further studies could define which chemical compounds detected in the studied essential oil may be responsible for the observed potential.

Regarding the antiproliferative activity, it was demonstrated the capacity of *E*. *pyriformis* essential oil to inhibit the proliferation of all four human tumor cell lines studied (Table 3). For the other, the essential oil did not present a significant capacity to inhibit the proliferation of the non-tumor primary culture in the concentrations evaluated. The essential oil of *E*. *pyriformis* showed an SI greater than 1 (Table 3), demonstrating a higher selectivity for tumor cells than for non-tumor cells tested (Almeida et al., 2014).

Some natural compounds have been identified as protective against damage induced by various carcinogens, including tobacco smoke. In particular, caryophyllane sesquiterpenes have recently attracted great scientific interest due to their antimutagenic effects against nitroarenes and aromatic amines (present in tobacco smoke), as well as anti-inflammatory and antiproliferative activity in cancer cells (Di Giacomo et al., 2018).

Due to the therapeutic insights of sesquiterpenes, such as antitumor, anti-inflammatory, and antimicrobial effects, among others, herbal medicine researchers have notably examined the use of this class in the medication of different diseases (Abu-Izneid et al., 2020), many resulting from tobacco use. The sesquiterpene β-caryophyllene and its metabolite β-caryophyllene oxide present in the essential oil of *E*. *pyriformis* (Table 1) exhibit a polypharmacological profile characterized by blocking, suppressive, chemosensitizing, and cytoprotective properties, suggesting application as a chemopreventive agent. The compound β-caryophyllene oxide was reported to possess antifungal, genoprotective, antioxidant, anti-inflammatory, chemosensitizing, and antiproliferative properties (Di Sotto et al., 2020).

The ability of the sesquiterpene β-caryophyllene and its metabolite β-caryophyllene oxide to inhibit the genotoxicity of a cigarette smoke condensate was evaluated in mammalian cells by Di Giacomo et al. (2018). The results indicated that β-caryophyllene induced the greatest genoprotective effect, reaching 60% inhibition at a concentration of 5 μg mL^−1^, while β-caryophyllene oxide at 10 μg mL^−1^ achieved 40% inhibition of DNA damage, caused by cigarette smoke condensate. These authors also tested the effect of sesquiterpenes on human HepG2 liver cancer cells. Different concentrations of cigarette smoke condensate (50–300 μg mL^−1^), β-caryophyllene and β-caryophyllene oxide (1–100 μg mL^−1^) were tested for mitochondrial cytotoxicity by the MTT colorimetric assay [3- (4,5-dimethyl-2-thiazolyl)-2,5-diphenyl-2H-tetrazolium]. A reduction in cell viability was observed by both sesquiterpenes at 100 μg mL^−1^, with an inhibition value of 70% for beta caryophyllene and 55% for Caryophyllene oxide. And in this sense, Kishi et al. (2022) reported that the addition of β-caryophyllene to cigarrettes may lower the risk of adverse health effects in passive and active smokers. The results found by these authors indicated that inhalation of betacaryophyllene in mice prevented the degradation of elastin fibers induced by nicotine and increased the expression of matrix metalloproteinase-2 (MMP-2) in the aortic wall.

The most abundant sesquiterpene in the essential oil of *E*. *pyriformis* in this study was spathulenol (45.6%) (Table 1). Studies of essential oils containing spathulenol as a major compound have reported some biological activities, such as antiproliferative, anti-inflammatory, and antimicrobial activities. And in this sense, do Nascimento et al. (2018) investigated anti-inflammatory, antiproliferative activity against MCF-7 and OVCAR-3 cell lines of essential oil extracted from *Psidium guineense* (Myrtaceae) leaves. The results indicated the presence of 80.7% spathulenol. The anti-inflammatory effect of essential oil was evaluated through the carrageenan-induced mice paw edema and pleurisy model. Essential oil (100 mg kg^−1^) was administered orally, observing maximum inhibition of 56.50% after 4 h of carrageenan administration. These same authors also investigated the anti-inflammatory effect of spathulenol in a pleurisy model that induces inhibition of inflammatory parameters such as leukocyte migration and protein extravasation. The results were expressed as leukocyte count and plasma extravasation in mg protein mL^−1^. Administration of carrageenan into the pleural cavity of animals induced an increase in the total number of leukocytes and an increase in protein extravasation 4 h after injection. Spathulenol (100 mg kg^−1^) administered orally significantly reduced the increase in total leukocytes (inhibition of 75.20%, and reduced the increase in protein levels (inhibition of 82.00) induced by carrageenan in the pleural cavity.

dos Santos et al. (2022) also investigated the anti-inflammatory and antinociceptive effects of spathulenol present in the essential oil of *Psidium guineense* (Myrtaceae) leaves. Pure spathulenol was used orally in male Swiss mice at a dosage of 10 mg kg^−1^, which was also administered locally (1,000 µg/paw^−1^). The results indicated that spathulenol managed to inhibit mechanical hyperalgesia by 76.00%, reduced the response to cold by 71.90%, and promoted the reduction of paw edema by 85.00%, thus indicating the anti-inflammatory and analgesic effect of spathulenol.

It is worth highlighting in this discussion the research with iridoid terpenes that demonstrated cellular protective effects against the effects of tobacco, such as those reported by Schmieder et al. (2007). These authors selected 22 natural extracts of *Gentiana lutea* (Yellow Gentian), as well as fractions containing pure isogentisin (1,3-dihydroxy-7-methoxyxanthone) and evaluated the potential to protect human vascular endothelial cells against cell damage induced by cigarette smoke. The cells were exposed for 4.5 h to chemicals in cigarette smoke; and then, the compound isogentisin was added to the cells, verifying the protection of endothelial cells from cell death. The authors performed detailed analyses of intracellular oxidative stress and protein oxidation and suggested that isogentisin promotes cell survival by activating cellular repair functions.

Sesquiterpenes are rapidly bioavailable through pulmonary, oral, and dermal administration, can effectively penetrate the blood-brain barrier and are generally recognized as safe for human health and have biological potential, such as β-caryophyllene and its metabolite caryophyllene oxide, which have anti-inflammatory, antioxidant and cytoprotective effects (Laws III and Smid, 2024). However, monoterpenes have a high cytotoxic potential demonstrated in several organisms, such as α-terpineol and terpinolene, which are among the most toxic terpenes, along with humulene and linalool. The IC_50_/LC_50_ or LD_50_ value for each terpene is variable. It depends on time and dose, where the cytotoxic effect is mainly caused by rupture of the plasma membrane or lipid peroxidation, or by the production of reactive oxygen species (ROS), loss of mitochondrial trifunctional protein (MTP), and mitochondrial impairment. In this sense, the most affected organs are the liver, kidney, lungs, and neurological tissues (Agus, 2021).

The absence of the ability of the essential oil to inhibit non-tumorous PLP2 cells (Table 3) indicates the absence of cytotoxicity of the oil. In this sense, there would be a doubt whether the main components, spathulenol and caryophyllene oxide, also present low cytotoxicity. In this sense, Di Sotto et al. (2020) reported in their studies that β-caryophyllene oxide does not present a genotoxic risk, despite the presence of a potentially dangerous epoxide group in its structure. Epoxides are not all equally dangerous and their reactivity can be affected by several factors. The epoxide function of β-caryophyllene oxide indicates that it is the only reactive site in the molecule and is included in an inflexible structure with a vicinal ethyl group that can hinder its reactivity by releasing electrons. Furthermore, the epoxide ring can open in the biological environment, thus forming derivatives that are not reactive to DNA. All these structural characteristics may justify the lack of genotoxicity of β-caryophyllene oxide.

In this context, the low cytotoxicity of *E. pyriformis* essential oil can be justified by the absence of toxicity of the main compounds of the essential oil spathulenol and caryophyllene oxide. This result opens new perspectives for continuing the research to prove the biological properties, for possible applications of the essential oil from *E. pyriformis* leaves in the food and pharmaceutical areas.

This study can be considered as a preliminary approach that should be complemented by exploring other parts of the plant such as flowers, fruits and leaves at different phenological stages, comparing with standard compounds.

## 5 Conclusion

The essential oil extracted from the leaves of *E. pyriformis* presented oxygenated sesquiterpenes as the majority class, with spathulenol and caryophyllene oxide as the main compounds. The essential oil showed antimicrobial activity against all microorganisms tested, with the most interesting activity against the bacteria *L*. *monocytogenes* and *S*. *Typhimurium*, and the fungi *P*. *ochrochloron* and *A*. *ochraceus*. The essential oil also exhibited the capacity to inhibit the production of the pro-inflammatory mediator nitric oxide and the proliferation of all tumor cell lines tested. The anti-inflammatory and antimicrobial activities observed in this study and complemented by the antifungal, genoprotective, antioxidant, chemosensitizing, and antiproliferative actions reported in the literature suggest that the essential oil, as well as the major compounds, spathulenol and caryophyllene oxide, may have a protective effect against damage caused by tobacco.

## Data Availability

The raw data supporting the conclusion of this article will be made available by the authors, without undue reservation.
